# Assessing the reliability and degradation of 10–35 years field-aged PV modules

**DOI:** 10.1371/journal.pone.0261066

**Published:** 2022-01-19

**Authors:** Muhammad Noman, Shanshan Tu, Shahab Ahmad, Fahad Ullah Zafar, Haseeb Ahmad Khan, Sadaqat Ur Rehman, Muhammad Waqas, Adnan Daud Khan, Obaid ur Rehman

**Affiliations:** 1 U.S.-Pakistan Center for Advanced Studies in Energy, University of Engineering & Technology, Peshawar, Pakistan; 2 Engineering Research Center of Intelligent Perception and Autonomous Control, Faculty of Information Technology, Beijing University of Technology, Beijing, China; 3 Department of Electrical Engineering, University of Engineering and Technology, Mardan, Pakistan; 4 Department of Computer Science, Namal Institute, Mianwali, Pakistan; 5 Department of Electrical Engineering, Sarhad University of Science & Information Technology, Peshawar, Pakistan; Xiamen University, CHINA

## Abstract

The objective of this study was to conduct a reliability analysis on photovoltaic (PV) modules from the oldest PV installation site in Pakistan. Four sets of modules; Type A & B (30 years old), Type C (10 years old), and Type D (35 years old) were identified for this analysis. It has been observed that modules have shown degradation after working for a good number of years in the field. Comparing with nameplate data (available for Type B & C only), a drop of 28.68% and 2.99 percentage points (pp) was observed in the output power (Pmax) and efficiency (Eff.) respectively for Type B, while a drop of 22.21% and 4.05 pp was observed in Pmax and Eff. respectively for Type C. A greater drop in I_SC_ and Pmax was observed in Type B, which is attributed to severe browning of EVA in them. While the greater drop in Pmax, in case of Type C, is attributed to the poor quality of materials used. Amongst the different defects observed, the junction box defects which include cracking and embrittlement, etc., and backsheet defects which include discoloration, delamination and cracking, etc. were found in all four types of modules. Other defects include browning of EVA, observed in Type B and D, and corrosion of frame and electrical wires, found in Type A, B, and D. This first-ever study will provide valuable information in understanding the degradation mechanism and henceforth, improving the long term reliability of PV modules in the humid-subtropical conditions of Pakistan.

## Introduction

The use of photovoltaic (PV) modules throughout the world is increasing exponentially. This is because of abundantly and freely available solar energy resource, the environmental friendly nature of PV module, and its ability to be used both as off-grid and on-grid systems [1–6]. Amongst the different renewable energy resources, currently, solar energy is the third most widely used resource in the world. In the last decade, the globally installed capacity of photovoltaic modules has increased from 70 GW in 2011 to more than 750 GW in 2020. The total PV installations were greater than 100 GW in the years 2017, 2018, 2019, and 2020 [7–10]. Pakistan is situated on an ideal location for solar energy applications and can exploit the abundantly available solar energy to fulfill its energy needs. The average global horizontal irradiation (GHI) is 200–250 W/m^2^ per day with 1500–3000 sunshine hours per year. The mean daily solar power potential of the country is 5.3 KWh/m^2^. In the overall energy mix of the country, for renewable energy, Pakistan plans to have a share of around 9.7 GW by 2030. To fulfill this goal, Pakistan installed its first solar-based on-grid plant in 2010 having a capacity of around 178 kW. A 1000 MW solar power project has also been initiated in Bahawalpur (Punjab) named “Quaid e Azam Solar Park”, whose current installed capacity is 400 MW [11–13].

The reliability and lifetime of PV modules depend upon their construction, the manufacturing process as well as the environment in which they are installed. The PV modules undergo degradation, such as EVA discoloration, module delamination, module glass and cell breakage, junction box damage and corrosion of metallic parts, etc. All these degradative losses, which are caused by the high humidity levels, ultraviolet radiations, extreme temperature cycles, wind, snow, hail, dust, and high system voltages, lead to a drop in the output power, efficiency, and the reduction in the useful life of the modules. Researchers are of the view that PV modules would be valuable only if they have a field life of at least 20 to 25 years of undiminished performance [14–22]. Therefore, to ensure reliability, the International Electro-technical Commission (IEC) has developed international standards under which modules are tested. These tests simulate the conditions which the modules undergo during their lifetime to evaluate their performance by accelerated aging methods [23]. However, even modules qualified under these standard tests tend to degrade before their predicted time. Thus, there is a great need for outdoor study and examination of the PV modules installed in the field. This will help in predicting the lifetime of PV modules properly and designing better products, by recognizing the failure mechanisms of the modules and their causes. Outdoor study of the PV modules, however, is a difficult job to perform as it involves several complications. It always carries a great loss to dismantle a running and functional system for the examination. That is why the defects in the modules and the operational shortcomings of the PV systems are important to be detected at an early stage [3, 14, 24].

The objective of this study was to analyze the degradation and the performance of the PV modules exposed to the outdoor environment for 10 to 35 years using different characterization techniques and recognize the failure modes in the humid-subtropical climate of Pakistan.

## Methodology

Four different types of PV modules were identified for this study, installed in the humid-subtropical climate conditions of Islamabad, Pakistan (latitude: 33.71° N & longitude: 73.10° E [25]) at two different places. A total of 12 PV modules, 3 from each type, were randomly selected which were a subset of 90 modules installed on commercial sites at the location mentioned. The investigation on the degradation and performance of the modules was carried out using visual inspection, electroluminescence (EL) imaging, UV fluorescence (UV-F), and IV measurement tests [24, 26]. All the modules were made up of crystalline silicon cells with ethylene-vinyl acetate (EVA) as encapsulant and Tedlar as backsheet. Table 1 provides the details of the modules under investigation.

**Table 1:** Details of PV modules under investigation.

Identity of Module	Type of Cells (Crystalline Silicon)	Country of Origin	No. of Cells	Time in the field (years)	Modules Selected for Study
**Type A**	Circular & Poly	Pakistan	40	30	3
**Type B**	Square & Poly	Japan	44	30	3
**Type C**	Square with Cut Edges & Mono	China	32	10	3
**Type D**	Circular & Poly	France	72	35	3

The modules were installed on the rooftop and the site of installation of the modules was surrounded by a commercial area. Type A modules were manufactured locally in Pakistan while Type B, C & D were imported from different countries. The specifications of each type of module are given in Table 2.

**Table 2:** Nameplate ratings of the PV modules.

Identity of Module	Manufacturer Data*						
	I_sc_ (A)	V_oc_ (V)	P_max_ (W)	I_max_ (A)	V_max_ (V)	Eff. (%)	FF (%)
**Type A**	-	-	-	-	-	-	-
**Type B**	3.06	25.0	57.2	2.86	20.0	10.42	73.92
**Type C**	5.94	21.4	100	5.74	17.44	18.16	78.75
**Type D**	-	-	-	-	-	-	-

*The manufacturer data of the Type A and D modules was not available as they did not have the name-plates attached with them.

### A. characterization techniques

The characterization techniques used in this study are presented below.

### 1) Visual inspection

The visual inspection of the modules was carried out to check for any defects or damages through naked-eye using the checklist prepared by the National Renewable Energy Laboratory (NREL) USA [27]. This gives an account of any degradation in the module’s material and the physical defects that are caused because of the environmental conditions.

### 2) Electroluminescence (EL) tests

This test was performed using an electroluminescence setup (Model: GEL-610 manufactured by Wuhan Gobo Photoelectric Technology Co. Ltd., PRC) available at the test site, i.e. Pakistan Council of Renewable Energy Technologies (PCRET), Islamabad, Pakistan. Electroluminescence is an optical phenomenon in which the PV module is connected to a DC voltage source in forward biased mode and current equal to the module’s short circuit current is allowed to pass through it. As a result, photons are emitted by the module, in the IR region which are captured by charged coupled device (CCD) based IR camera. The intensity of the light emitted is dependent upon the density of minority charge carriers. Thus, any defect that changes the minority charge carriers’ density, can be detected using this technique. Due to the difference in the emitted light intensity, a variation in the brightness of the image is observed which gives an insight into the module’s structure and the defects present in it [28].

### 3) Ultraviolet fluorescence (UV-F) imaging

In this technique, ultraviolet light is made to fall on the module through a UV light source and the results are observed using any visual detecting tool. It is used to identify the defects that are not visible with the naked eye and has become an effective method to study encapsulant related issues. EVA which is the most commonly used encapsulant in the PV module forms fluorophores due to the exposure to UV rays present in the sunlight and high temperature. Fluorophores are species having the ability to emit light in the visible range when excited by UV light. This property of the fluorophores is used in UV-F imaging. Any difference in the intensity of the light emitted back can be used to analyze the defects. UV-F can also be used for detecting cracks in the cells and hot spots in the PV modules [29–33]. UV-F imaging of the present modules was performed in a dark room to remove any effect of visible light.

### 4) Solar flash (I-V Curve) tests

Solar flash test is used to find out the electrical parameters of the PV module using a setup that simulates the solar intensity. This test was performed under standard testing conditions (STC) using AAA class solar flash tester (Model: GSMT-H-3A manufactured by Wuhan Gobo Photoelectric Technology Co. Ltd., PRC) available at PCRET, Islamabad [23]. Degradation of the modules was calculated by comparing the nameplate data with the data obtained through the solar flash test.

## Results and discussion

### A. visual inspection

A thorough visual inspection of the modules under study was carried out, for which a detailed checklist prepared by NREL was used [27]. As shown in Figs 4–6, 10, various defects and degradations were noted during this inspection, among which the most prominent were discoloration, delamination, burn marks and scratches at the backsheet; corrosion of the connecting wires and busbars; encapsulant discoloration; damages to the frame and the junction box (JB), and soiling of the front glass. These defects are divided into six major types i.e. backsheet defects, wires defects, frame defects, junction box defects, discoloration, and front glass soiling.

**Fig 1:**
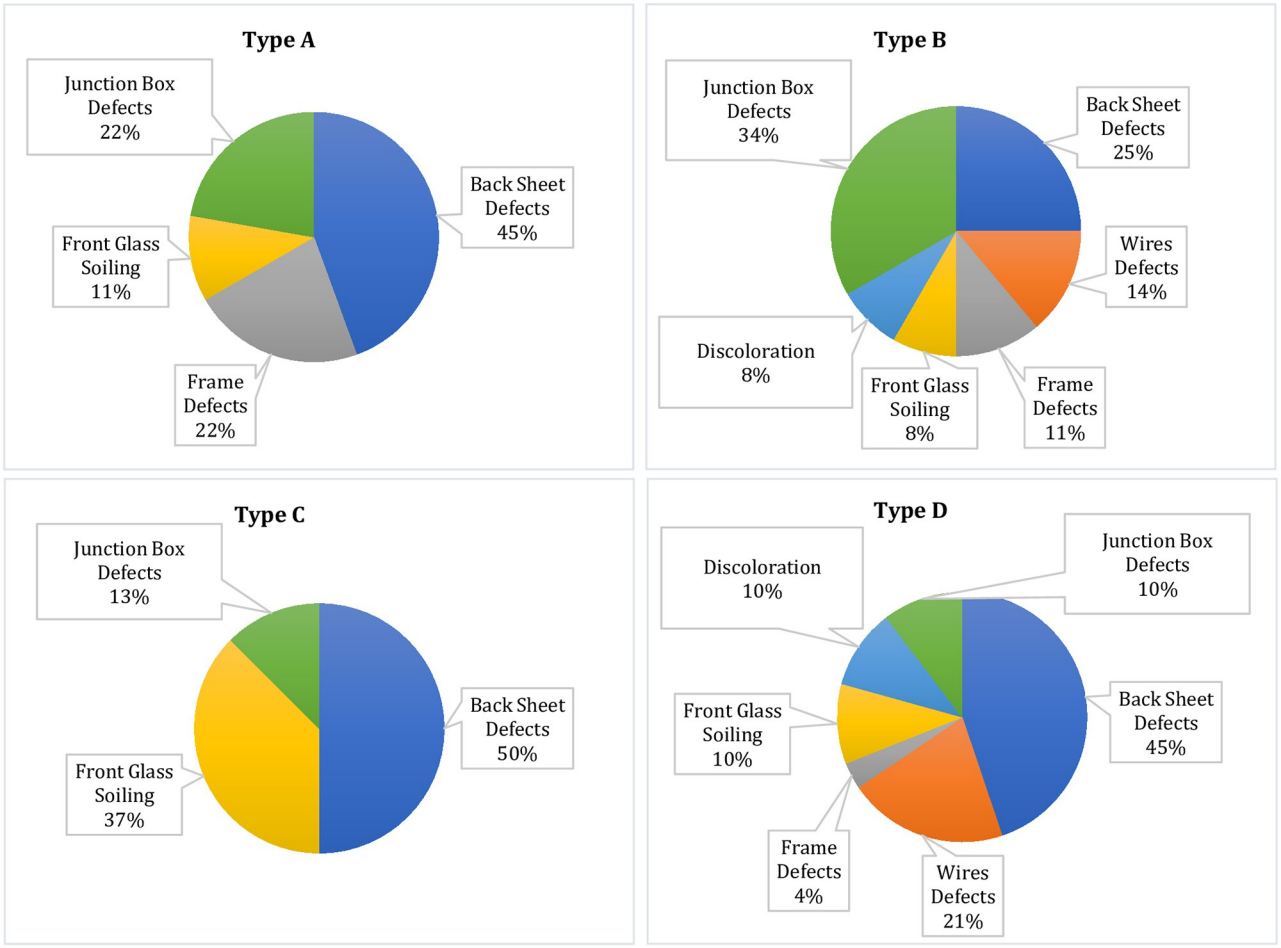
Percentage occurrence of different defects in the modules under observation.

Fig 1 shows different defects observed in all the four types of modules in the form of the percentage of their occurrence. The percentage occurrence helps us in the quantification of different defects observed during the examination process. To understand the percentage occurrence of the defects, we look at Type A modules in Fig 1, where the junction box defects are 22%, backsheet defects are 45%, frame defects are 22% and front glass soiling is 11%. It means that out of the total defects found in Type A modules, 22% were related to the junction box, 45% to backsheet, 22% to frame, and so on. Thus, the greater the percentage of a defect type, the greater is its presence in the modules.

**Fig 2:**
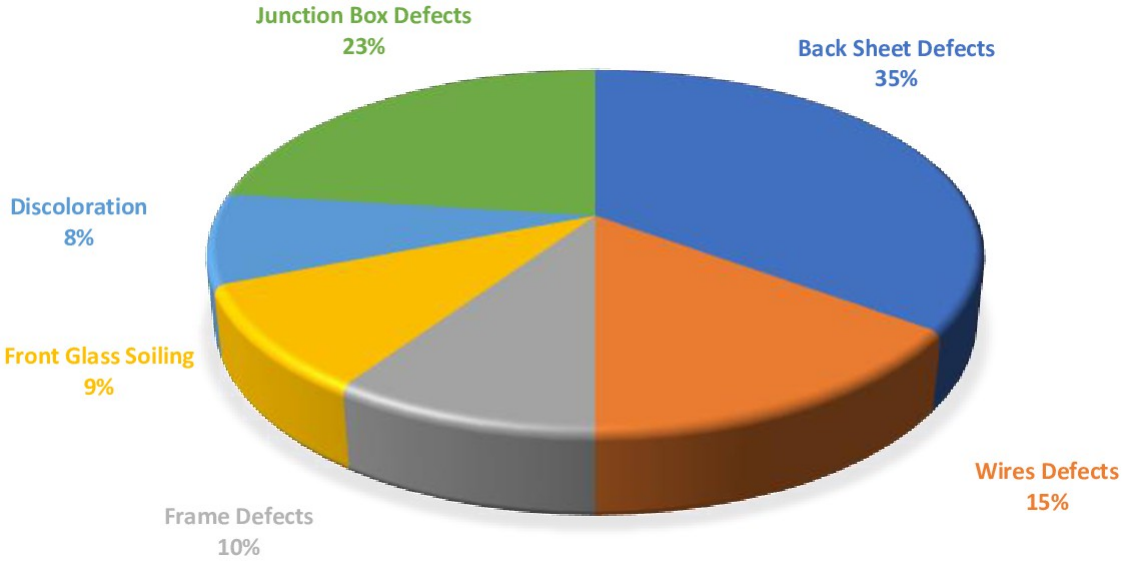
Percentage occurrence of defects (combined) in Type A, B & D modules.

Type C modules that were 10 years old showed three types of defects. All the other modules (Type A, B & D) have been in the field for almost the same time, so the defects in these modules were collectively analyzed. Fig 2, shows the percentage of different defects observed in Type A, B, and D modules when they are combined. The different defects observed during the visual inspection of the modules are discussed below.

**Fig 3:**
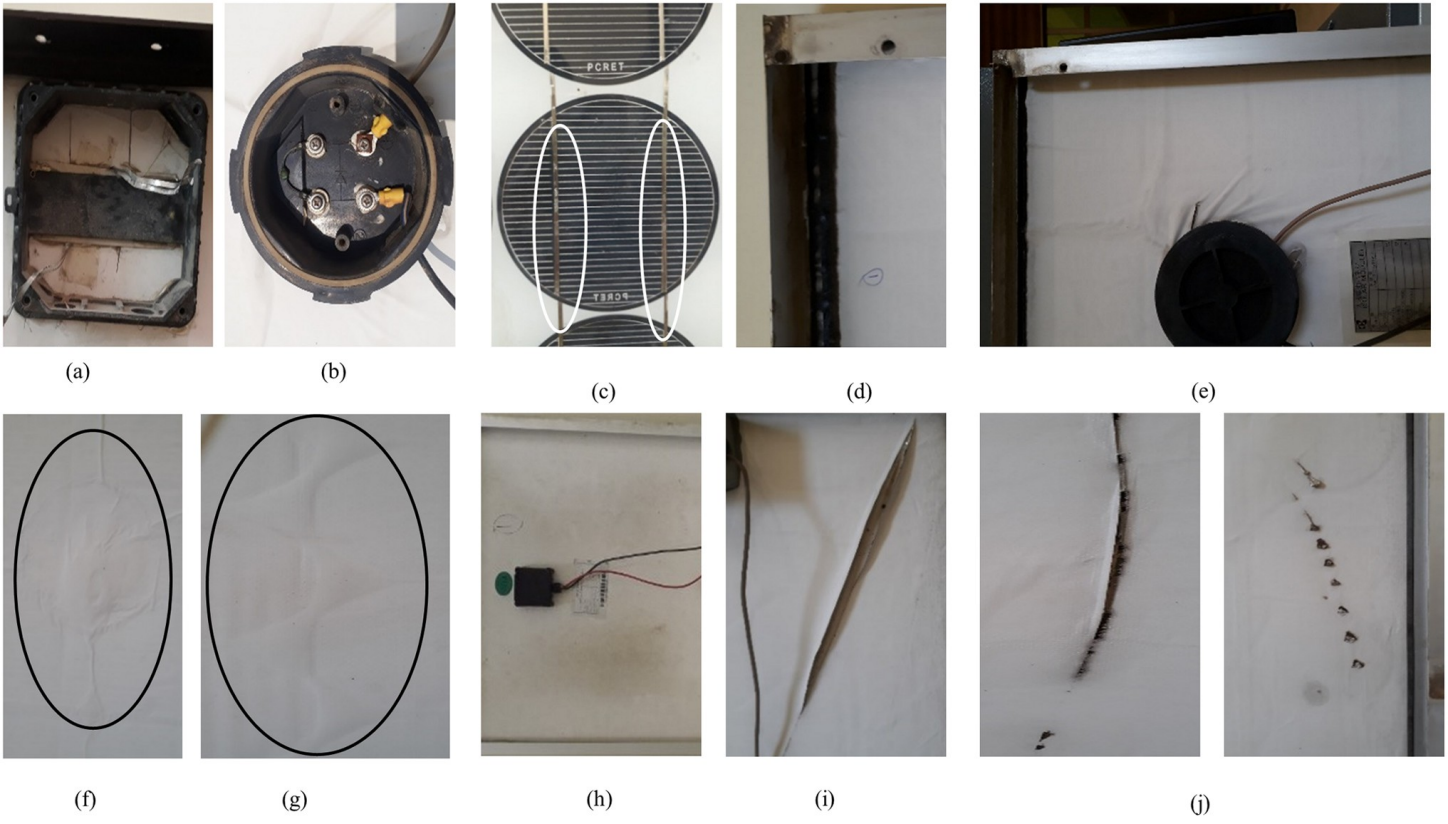
Defects observed in visual inspection: (a) & (b) Broken junction box and corroded wire in Type A & B respectively, corrosion of (c) busbars (Type A) & (d) metallic frame (Type B), (e) & (f) delaminated backsheet in Type B & (g) Type D, (h) discoloration of the backsheet (Type C), (i) & (j) scratch/crack & burn marks on backsheet in Type D.

The percentage occurrence of backsheet defects is greatest in all four types of modules. The defects observed in the backsheet include discoloration, delamination & bubbling, slightly chalking (formation of a white powder), scratches/cracks, and burn marks. The discoloration of the backsheet is caused by the short-wavelength radiations present in the solar spectrum, which chemically degrade the backsheet when incident on it. The backsheet is generally made up of three layers bonded together using an adhesive. Exposure to extreme humid environment causes the loss of adhesion properties of the adhesive which results in delamination of the backsheet. In addition to this, the moisture or atmospheric gasses can enter into the laminate through the permeable and/or degraded backsheet and get trapped due to different permeation properties and give rise to bubble formation. The chalking is originated from the photothermal degradation of the outer surface of the backsheet, which brings the internal additives to the surface and causes its easy abrasion. Scratches or cracks are the results of the loss of adhesion of the backsheet due to its degradation caused by high humidity levels. When the outer layer of the backsheet is delaminated, it gives rise to high internal residual stresses and thus the outer layer is more vulnerable to cracking in a high humid environment [34–36]. Amongst the different modules used in this study, the backsheets of the Type C modules were slightly chalked and discolored which is because of their photothermal degradation. On the other hand, the backsheets of Type A, B, and D modules exhibited the different defects mentioned above. This is because these modules were exposed to an extreme humid environment throughout their life (30+ years) which caused the delamination, cracks, and bubbling of the backsheet in them. The JB of the Type C modules was only slightly damaged while all the other types of modules showed various defects related to JB. These defects include cracking, warping, embrittlement, and weakening of adhesion. Moreover, the JB of some modules had loose lids while some did not have any lid attached with them. The wire defects were seen in Type A, B, and D modules only, as the JBs of the Type C modules were intact, thus they protected the connecting wires from degradation. In the former types, the connecting wires of some of the modules were corroded and some did not have any connectors attached to them. Another defect observed during the visual inspection was the frame defect, found in Type A, B, and D modules. The frame of some of these modules was loosely attached (weakening/loss of adhesion) while other modules showed corrosion of some part of the frame. The representative images of the defects observed during the visual inspection are shown in Fig 3.

**Fig 4:**
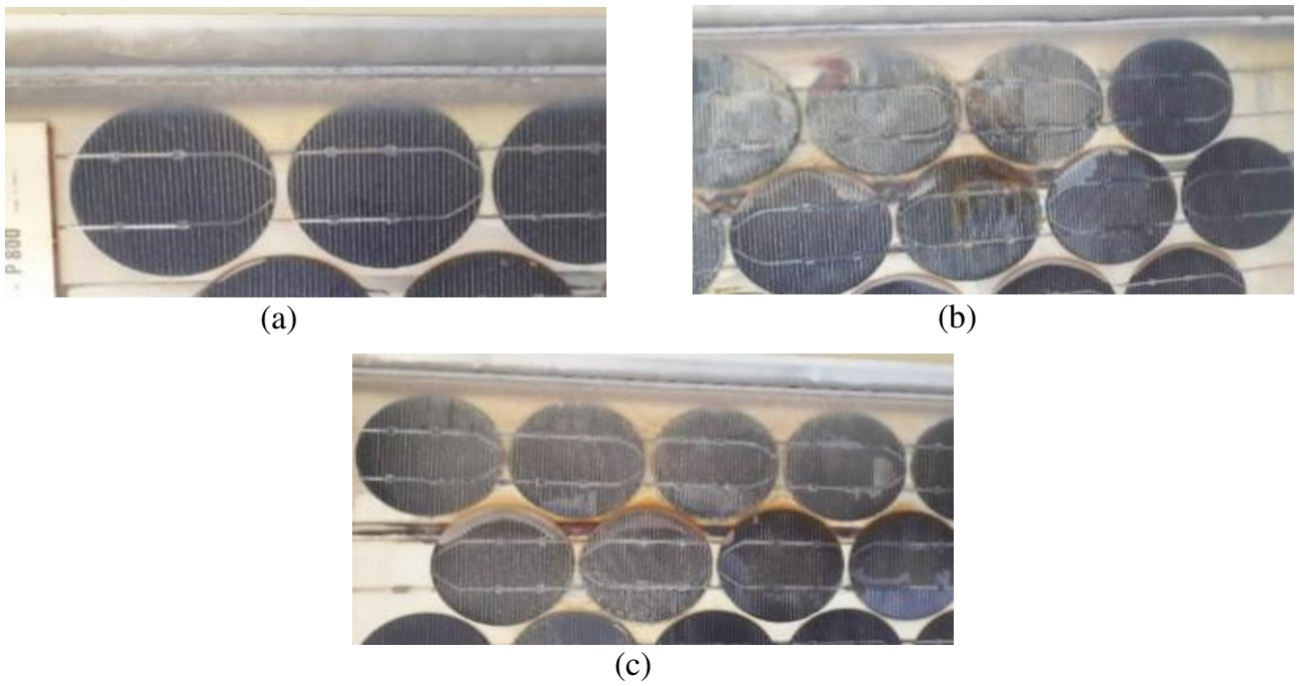
Visual inspection of Type D modules showed: (a) discoloration near the cell edges, (b) & (c) discoloration of EVA & burn marks on the Si-cells.

Another issue commonly noticed in the field-installed modules is of soiling of the front glass, which was also found in all the types of modules during this study. Soiling of the front glass impacts the modules negatively in several ways. Firstly, soiling reduces the transmission of the light to the cells, thus the short circuit current decreases which reduces the power output of the module. Secondly, if the soiling over the module is non-uniform, it can produce partial shading effects and the mismatch of the electrical parameters of the shaded cell with the other cells of the module occurs, resulting in an increased risk of damage to the module. Moreover, the corrosion of the busbars and burn marks on the cells were also found during the visual inspection. The corroded busbars of the Type A module are shown in Fig 3(c) (encircled in white) and those of Type B are shown in Fig 6 (encircled in yellow) while the burn marks found in the Type D module can be seen in Fig 4.

**Fig 5:**
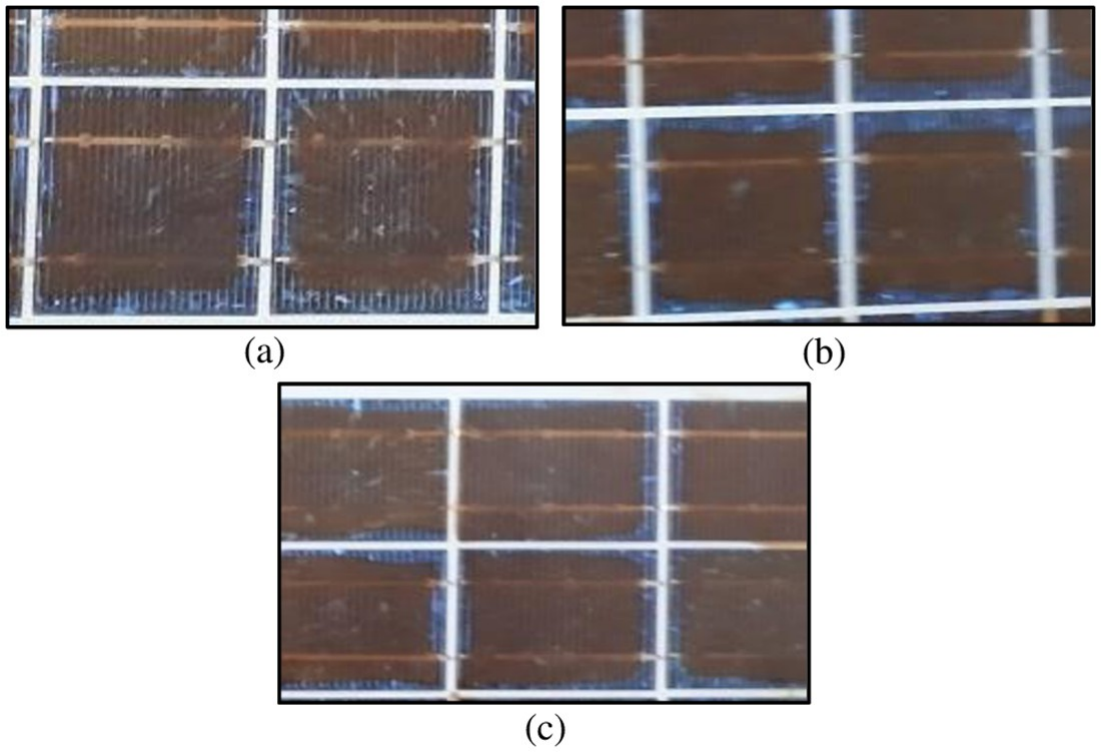
Discoloration of Type B PV Modules (effect of photo-bleaching is visible) in: (a) Module B (1), (b) Module B (2) & (c) Module B (3).

Type B and D modules showed all six types of defects, one of which was the discoloration of the encapsulant: ethylene vinyl acetate (EVA). During the production of EVA, certain additives are added to it which include curing agents, UV absorbers, UV stabilizers, and anti-oxidants. EVA is degraded (discolored) due to the high dose of UV light (in the solar spectrum) and high temperature that the module receives while working in the field. Different levels of discoloration have been reported in the literature, ranging from light yellow to yellowish-brown and dark brown with the greatest loss in the performance relating to the greatest discoloration [37–41]. In this study, Type D modules showed a light yellow to yellowish-brown discoloration (Fig 4) while dark brown discoloration was observed in Type B modules (Fig 5).

The discoloration (or browning in severe case) is a result of the chemical reactions between different ingredients of EVA in the presence of UV light at high operating temperatures. These reactions result in an increase in the cross-linking in EVA and the formation of polyenes and/or acetic acid and other volatile compounds. Polyenes are species containing unsaturated chromophores which cause browning of EVA. The color of EVA defines the extent of degradation in EVA: the darker the color of EVA, the greater is its degradation and the longer the chain of polyene. Besides these reactions, other chemical reactions also take place in the PV modules having a breathable/permeable backsheet. Oxygen molecules can enter into the module through a permeable backsheet and cause photo-bleaching of EVA, which is the oxidation of the long-chain polyenes in the presence of light. Photo-bleaching reduces the chain length of the polyene and transforms the brown EVA to a yellowish/whitish color [30, 38–43].

During the visual inspection, the encapsulant of Type B modules exhibited dark brown color, displaying severe degradation in them which is shown in Fig 5. The browning was greatest closer to the center of the cells and almost no browning was observed near the edges or in the region between the cells, making a square pattern over the cell. This is because of photo-bleaching over the edges or the regions between cells due to the diffusion of oxygen into the module (Fig 5). Similar effect has been reported in the literature previously [29, 32, 33, 37, 38].

**Fig 6:**
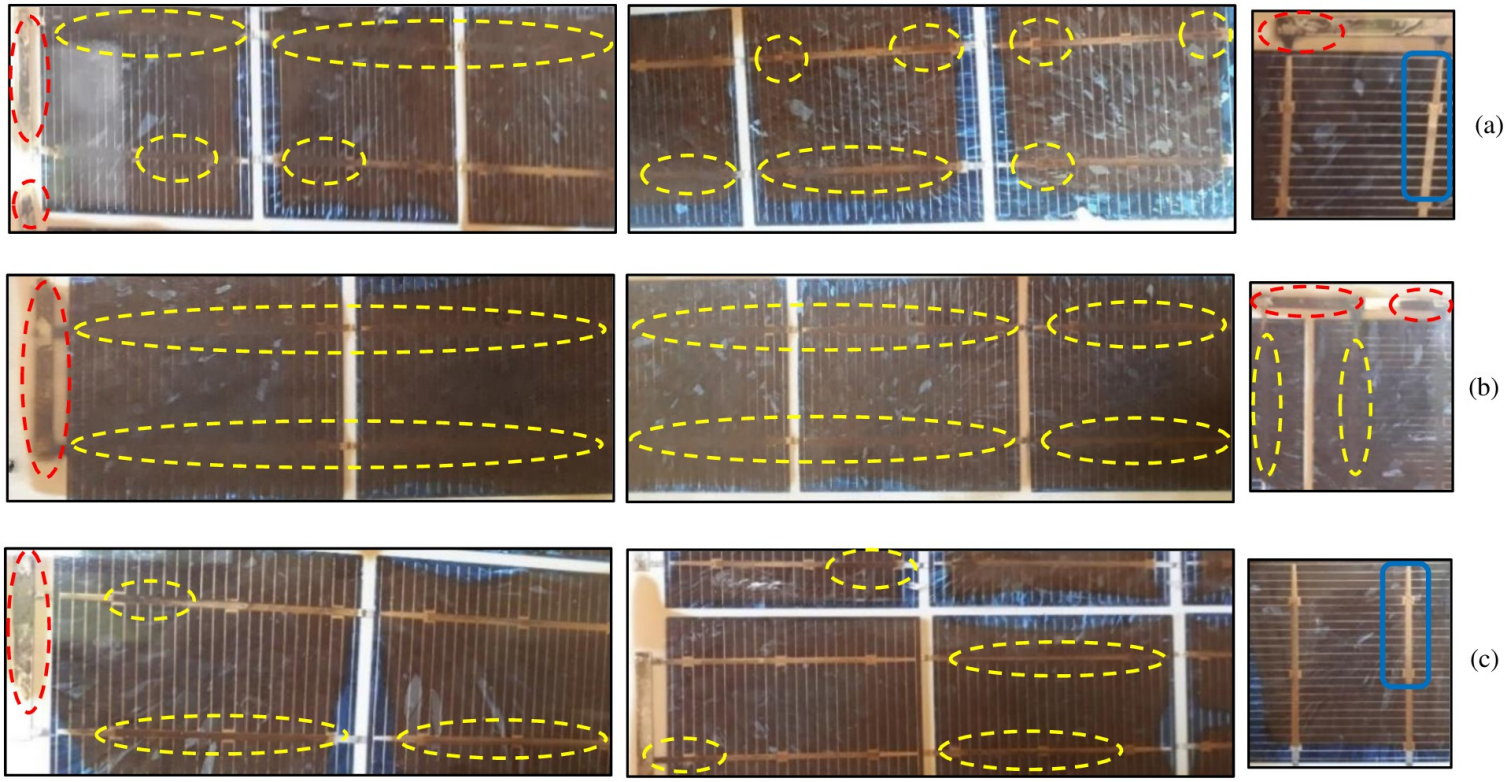
Corrosion of the metallic parts in Type B: (a) Module B (1), (b) Module B (2) & (c) Module B (3).

Browning of EVA negatively impacts the power output and short circuit current of the module by decreasing the light reaching the solar cell [30, 39, 40, 42]. This was also observed in Type B modules which showed a 26.18% drop in the short circuit current, as can be seen in Fig 9. Browning also affects the module indirectly by degrading other components alongside. The acetic acid and other volatile products of EVA degradation can cause the delamination effect in the module [39, 42–44]. This was observed during the visual inspection when the delamination of the backsheet was found in Type B and Type D modules only (shown in Fig 3). Moreover, along with the browning of the EVA, corrosion of the metal conductors and burn marks were also observed in the Type B and D modules. The corrosion of the busbars and copper (Cu) ribbons in Type B modules is shown in Fig 6. The reason for this corrosion is that the acetic acid in combination with the moisture coming into the module attacks the metallic parts and corrodes them [39–42]. The corrosion of the busbars is shown in yellow dashed circles while that of Cu ribbons can be seen in red dashed circles. For comparison purposes, images of the portion of the modules where busbars were not corroded are also shown in Fig 6. Greater discoloration was observed where busbars were corroded as compared to those where busbars were not corroded (blue rounded rectangle). In case when only a portion of the encapsulant gets discolored, it can produce the partial shading effect and thus cause hotspot generation due to the mismatch within the module [19, 42]. This was observed in Type D modules, where only a portion of EVA showed discoloration (Fig 4). In this case, the cell covered by the discolored EVA produces lesser current than the other cells in the module which results in reverse biasing of the shaded cell. Due to this mismatch, the covered cell gets heated up and ultimately burns. Fig 4 shows the burn marks on the cells in the Type D module.

### B. defects causing safety hazard

The defects observed in the visual inspection can cause safety issues like electrical, physical, and fire hazards. The defects found in the backsheet like burn marks, cracks or scratches, and delamination can cause electrical hazards. Discoloration (or browning) of the EVA and corrosion of the metal strings observed in the modules have the tendency to cause electrical, fire, and physical damage. The different defects observed in the JB and connecting wires may cause electrical, physical, and fire hazards [18]. Interestingly, all these defects which have safety hazards associated with them, are the highest occurring defects in the modules studied, as seen from Figs 1 and 2. Moreover, it was observed that one defect may lead to another; as discussed above, the EVA browning caused the corrosion of busbars and the generation of hotspots in the module. Also, the defective backsheet can become the root cause of other failure modes in the modules; when the delamination occurs near the junction box (Fig 3(e)) or the frame, water may enter into the module during the heavy rainy season or when there is dew, and cause corrosion of the busbars. Thus it can be inferred, that the corrosion of the busbars and Cu ribbons observed in Type B modules (Fig 6) occurred due to the degradation of the EVA and backsheet. Moreover, the corrosion of the busbars observed (Fig 3(c)) is due to the ingress of moisture into the module.

### C. electroluminescence (EL) imaging

**Fig 7:**
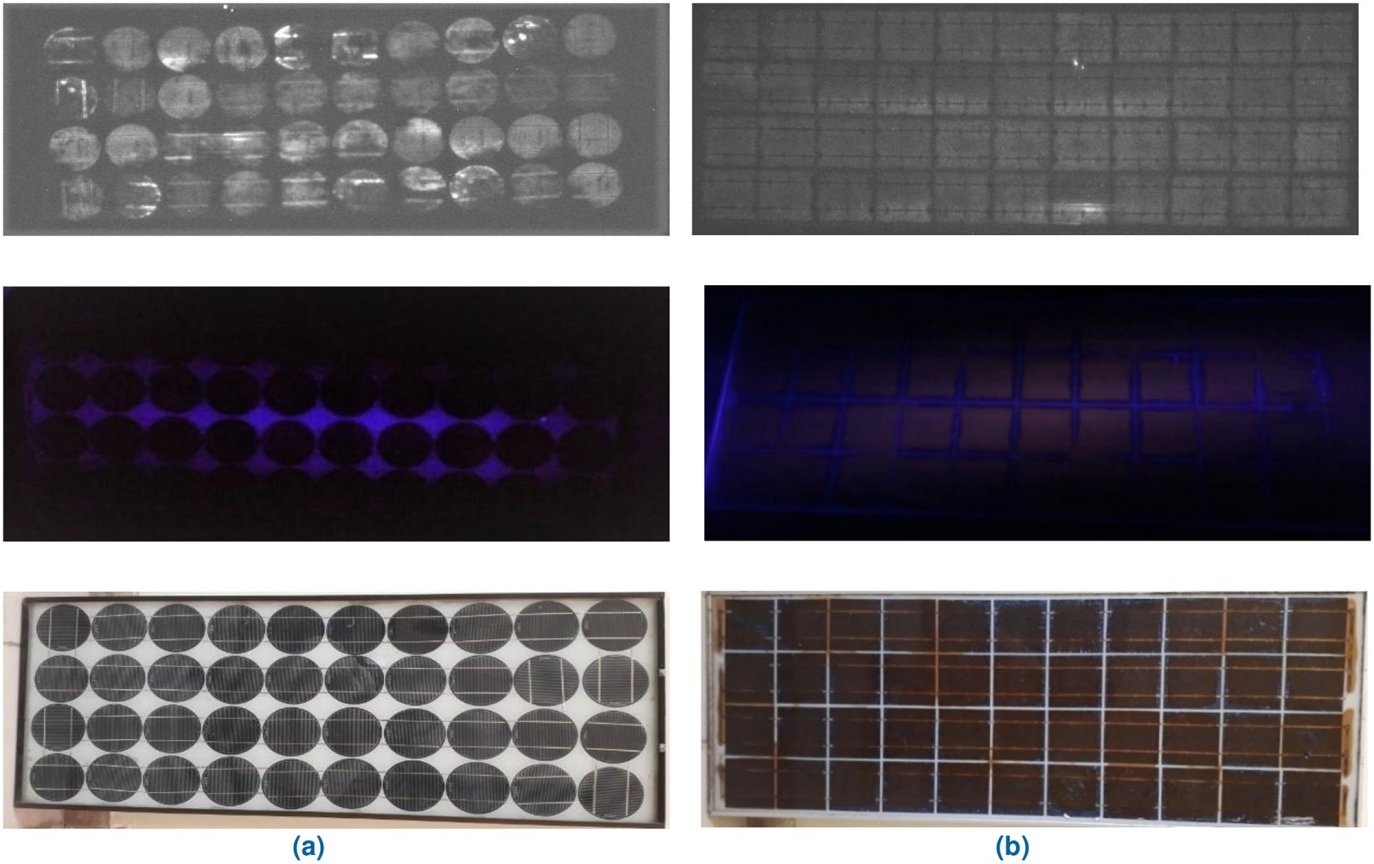
EL, UV-F and VL images of (a) Type A & (b) Type B modules.

**Fig 8:**
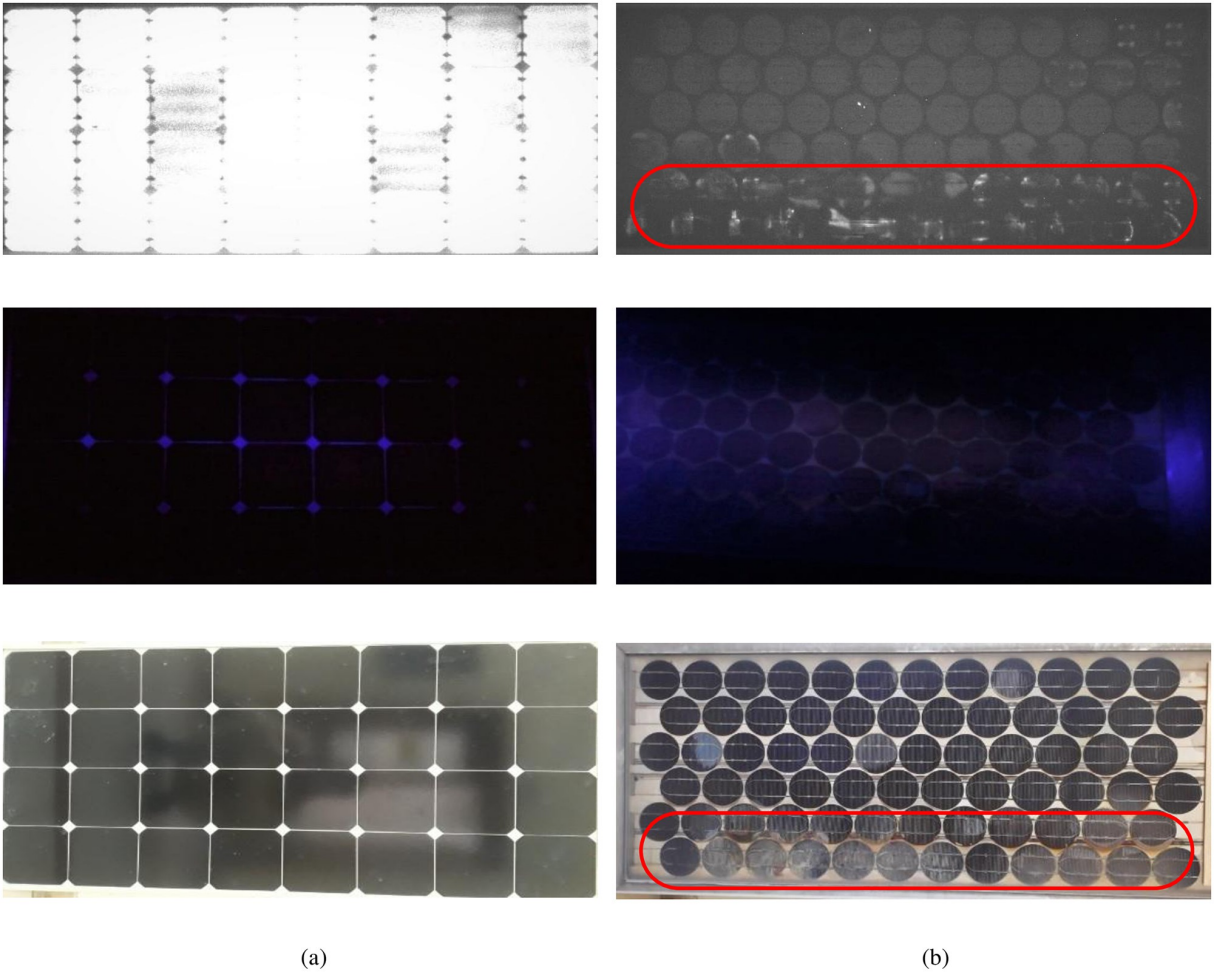
EL, UV-F and VL images of (a) Type C & (b) Type D modules.

The electroluminescence (EL) and visible light (VL) images of the modules under observation are presented in Figs 7 and 8. The EL image detects the defects in the module, which cannot be seen through the naked eye, as a mismatch in the brightness (lighter and darker regions). The dark areas of the EL image represent the defective parts of the cell that do not take part in the current generation. Fig 7 shows such dark areas for both Type A and B modules. Moreover, an irregular pattern in the EL image of the PV module represents the degradation of the encapsulant and burning effect of solar cells. The portion encircled in red, shown in Fig 8(b), represents such a type of degradation in the Type D module. This type of defect arises due to several reasons which include reverse biasing of the cell or part of the cell, partial shading of the module, presence of non-uniformities, and any defect that causes shunting. All these factors contribute to the hot spot generation and cause the burning of the cell [18]. The discoloration of the EVA in the module is visible in VL images (Figs 4 and 8(b)). This discoloration causes reverse biasing which ultimately leads to burning.

### D. Ultraviolet Fluorescence (UV-F) imaging

**Fig 9:**
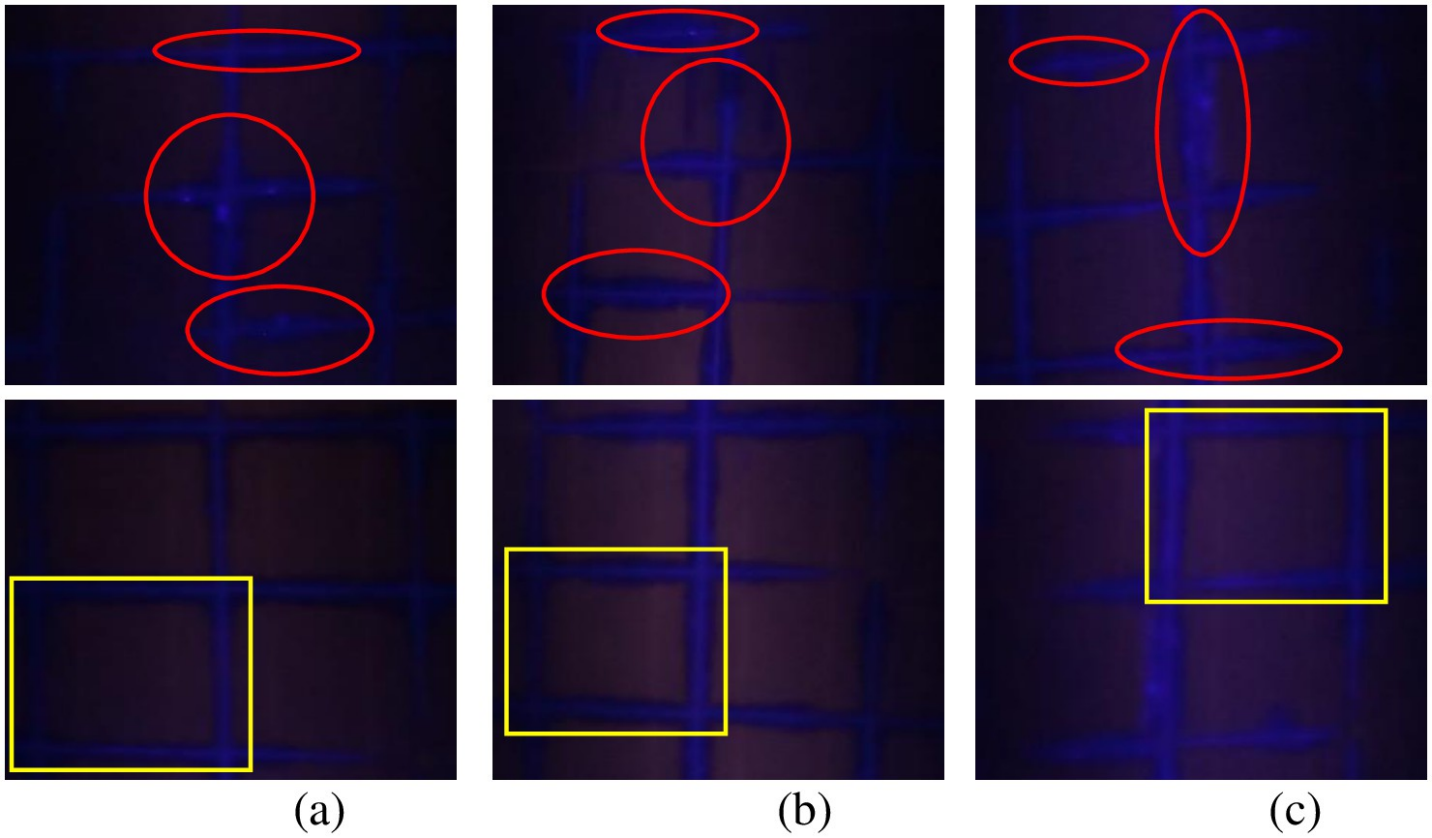
UV-F images of Type B modules (a) Module B (1), (b) Module B (2) & (c) Module B (3).

Representative results of UV-F imaging of the modules under observation are presented in Figs 7–9. The encapsulant discoloration observed during the visual inspection was also analyzed using UV-F imaging. By looking at the UV-F images of the Type B modules in Fig 9, the photo-bleaching effect (encircled in red) is quite evident. Moreover, Fig 9 also shows the square pattern (shown in yellow boxes) formed because of the competing reaction between the browning and the photo-bleaching of EVA.

### E. Performance degradation

**Fig 10:**
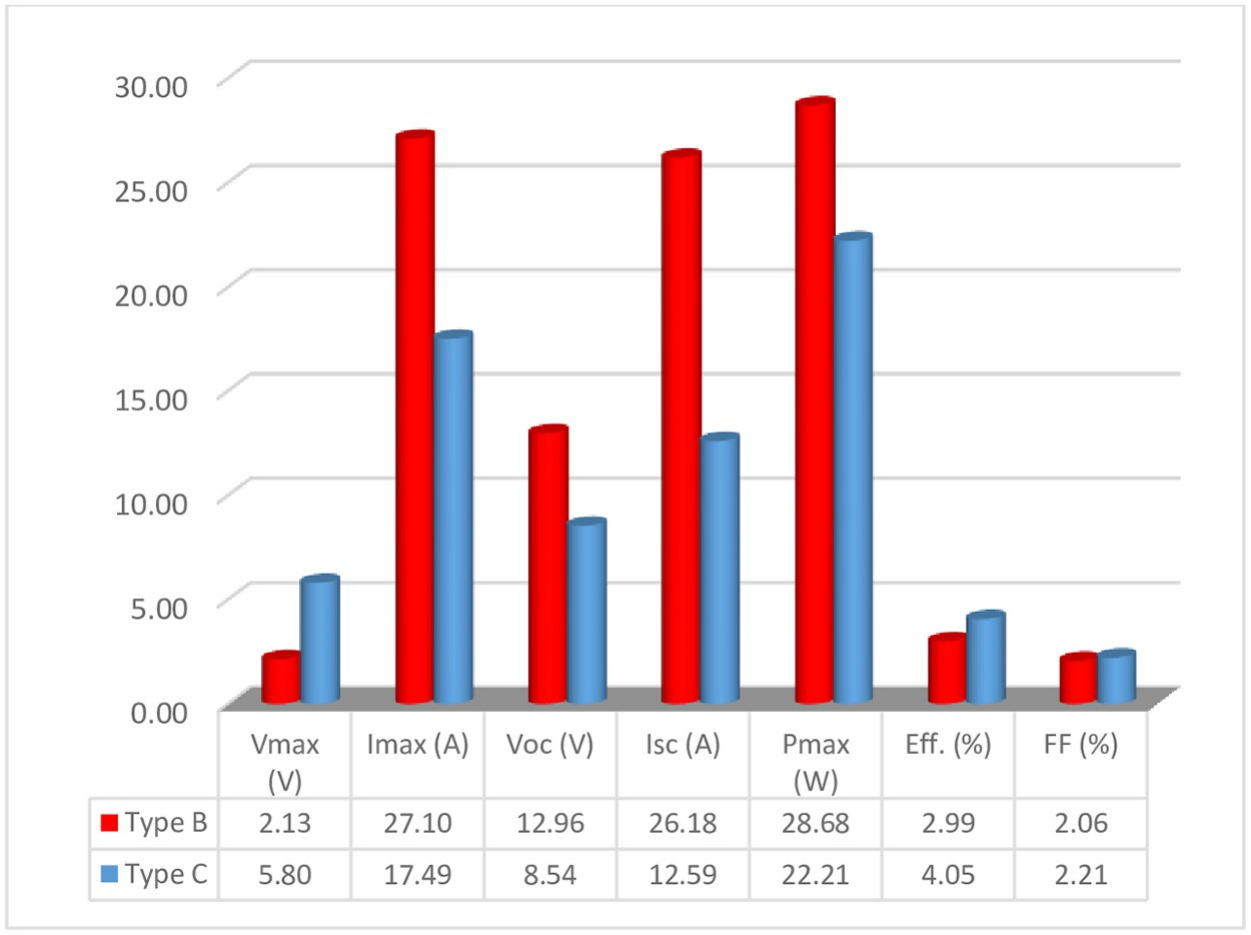
Percentage decrease in the performance parameters of Type B & Type C modules (Eff. & FF degradation is in percentage points).

The data obtained by the solar flash testing of the modules was compared with the nameplate data to evaluate the degradation in the performance parameters caused by different failure modes. Fig 10 shows the percentage decrease calculated for each parameter. The nameplate data for the Type A and D modules was unfortunately not available so the data of these modules could not be compared. The degradation in efficiency and fill factor are presented in percentage points (pp) in this paper.

It can be seen that after working for a good number of years in the field, the modules have shown some deterioration in the performance parameters. The power output (Pmax) and Efficiency (Eff.) have dropped by 28.68% and 2.99 pp respectively for the Type B modules, while for Type C modules, the Pmax has reduced by 22.21% and Eff. by 4.05 pp. A greater reduction in the short circuit current (ISC) and Pmax while only a slight drop in fill factor (FF) and open-circuit voltage (VOC) was found in the Type B modules which is evident from the percentage decrease in its parameters (Fig 10). This is attributed to the greater amount of browning of EVA in Type B modules. Browning reduces the transmission of light and increases its reflectance due to which greater reduction in the I_SC_ and Pmax while a very slight decrease in the FF and VOC was observed. Similar results have been previously reported in the literature [38, 39, 45]. Moreover, the Type B modules were old with an initial efficiency of 10% while that of Type C modules was 18%. Thus, a lesser decrease in the efficiency of Type B modules had a greater impact on their performance (as seen in Fig 10).

The power output after 10 years of field life for Type C modules is 77.79% which is lesser than the estimated power output (90%) for the modules after 10 years of field exposure [39]. This greater drop in the output power may be attributed to the poor quality materials used for the Type C modules. The percentage degradation per year in the electrical parameters of the Type B and Type C modules is shown in Table 3. Considering the percentage degradation/year in I_SC_ and Pmax of the Type C module which is 1.26 and 2.22%/year respectively. This degradation is very high as compared to the specified limit of the power output after 10 years of field life. Such a high degradation rate will not only reduce the power output of the degraded modules but can also reduce the un-degraded modules’ performance as well as the integrity. As obvious from Table 3, the percentage degradation/year in Type C modules is greater than Type B showing the poor quality of manufacturing/materials used in Type C modules.

**Table 3:** Percentage degradation per year in the electrical parameters of type B & C modules.

Parameter	Degradation per Year	
	Type B (%/year)	Type C (%/year)
**Vmax (V)**	0.07	0.58
**Imax (A)**	0.90	1.75
**Voc (V)**	0.43	0.85
**Isc (A)**	0.87	1.26
**Pmax (W)**	0.96	2.22
**Eff. (%)** *	0.10	0.41
**FF (%)** *	0.07	0.22

*For Eff. & FF, it is in percentage points per year

## Conclusion

Photovoltaic (PV) modules working in the field for 10–35 years in Islamabad, Pakistan were taken and studied for degradation and defects. Different types of defects were visible in the modules including a number of those which have the tendency to cause safety hazards. Out of the different failure modes identified during the visual inspection process, defects in the junction box and backsheet, and front glass soiling were found in all four types of PV modules. The percentage occurrence of these failure modes was 45%, 25%, 50% and 45% (backsheet defect) and 22%, 34%, 13% and 10% (junction box defect), 11%, 8%, 37% and 10% (front glass soiling) in Type A, B, C and D modules respectively. Type B and D modules showed 8% and 10% discoloration defects respectively, out of the total defects observed in them. This discoloration can cause corrosion of the busbars and copper ribbons which was observed in Type B modules and hotspot or burn marks in the modules as seen in Type D modules. The defects found in the modules during the visual inspection were easily visible when electroluminescence (EL) imaging and ultraviolet fluorescence (UV-F) techniques were used. The degradation in the performance parameters was calculated by comparing the results of the solar flash (I-V curve) test with the nameplate data available (Type B and C only). A decrease of 28.68%, 26.18%, 2.99 pp and 2.75 pp (Type B) and 22.21%, 12.59%, 4.05 pp, and 2.21 pp (Type C) in output power (Pmax), short circuit current (ISC), efficiency and fill factor (FF), respectively, was observed. The greater drop in ISC and Pmax while lesser in FF of the Type B modules is attributed to severe browning of EVA in them. The power degradation/year for Type B modules was 0.96%/year while for Type C modules it was 2.22%/year. The percentage degradation in the Pmax of Type C modules was found to be greater than the allowed limit for the degradation in the module’s power output after 10 years of field exposure which was attributed to the low quality of the materials/manufacturing process used for these modules. Although the qualitative damage appears serious, the quantitative results show that the modules were still producing power and the losses are not dramatic. Moreover, it was observed that a single failure mode is not only always responsible for the failure of the PV modules, but one failure mode may lead to another and thus cause degradation in the performance.

## Supporting information

S1 Data (XLSX)

S2 Data (XLSX)

## References

[1] IEA. Projected Costs of Generating Electricity– 2015 Edition. 2015; 215. https://www.oecd-nea.org/ndd/pubs/2015/7057-proj-costs-electricity-2015.pdf

[2] Kannan N, Vakeesan D. Solar energy for future world:—A review. Renew Sustain Energy Rev. 2016;62: 1092–1105. doi: 10.1016/j.rser.2016.05.022

[3] Kumar M, Kumar A. Performance assessment and degradation analysis of solar photovoltaic technologies: A review. Renew Sustain Energy Rev. 2017;78: 554–587. doi: 10.1016/j.rser.2017.04.083

[4] Kaewpraek C, Ali L, Rahman MA, Shakeri M, Chowdhury MS, Jamal MS, et al. The effect of plants on the energy output of green roof photovoltaic systems in tropical climates. Sustain. 2021;13: 1–10. doi: 10.3390/su13084505

[5] Sedaghat A, Abbas Oloomi SA, Malayer MA, Alkhatib F, Sabri F, Sabati M, et al. Effects of Window Films in Thermo-Solar Properties of Office Buildings in Hot-Arid Climates. Front Energy Res. 2021;9: 1–22. doi: 10.3389/fenrg.2021.665978

[6] Nwaigwe KN, Mutabilwa P, Dintwa E. An overview of solar power (PV systems) integration into electricity grids. Mater Sci Energy Technol. 2019;2: 629–633. doi: 10.1016/j.mset.2019.07.002

[7] Chowdhury MS, Rahman KS, Chowdhury T, Nuthammachot N, Techato K, Akhtaruzzaman M, et al. An overview of solar photovoltaic panels’ end-of-life material recycling. Energy Strateg Rev. 2020;27: 100431. doi: 10.1016/j.esr.2019.100431

[8] Jäger-Waldau A. Snapshot of photovoltaics—February 2019. Energies. 2019;12. doi: 10.3390/en12050769

[9] Jäger-waldau A. Snapshot of Photovoltaics—February 2020. 2020.

[10] Jäger-waldau A. Snapshot of photovoltaics À March 2021. 2021;2: 1–7.

[11] Mirza UK, Mercedes Maroto-Valer M, Ahmad N. Status and outlook of solar energy use in Pakistan. Renew Sustain Energy Rev. 2003;7: 501–514. doi: 10.1016/j.rser.2003.06.002

[12] Irfan M, Zhao Z-Y, Ahmad M, Mukeshimana M. Solar Energy Development in Pakistan: Barriers and Policy Recommendations. Sustainability. 2019;11: 1206. doi: 10.3390/su11041206

[13] AEDB—Pakistan. [cited 23 Jun 2021]. https://www.aedb.org/

[14] Sharma V, Chandel SS. Performance and degradation analysis for long term reliability of solar photovoltaic systems: A review. Renewable and Sustainable Energy Reviews. 2013. doi: 10.1016/j.rser.2013.07.046

[15] Da Silva Freire F. Performance and Degradation Analysis of Operating PV Systems. Thesis. 2016. http://gradworks.umi.com/10/14/10143004.html

[16] TamizhMani G, Kuitche J. Accelerated Lifetime Testing of Photovoltaic Modules Solar America Board for Codes and Standards. Sol ABC. 2013; 106.

[17] Ndiaye A, Charki A, Kobi A, Kébé CMF, Ndiaye PA, Sambou V. Degradations of silicon photovoltaic modules: A literature review. Sol Energy. 2013. doi: 10.1016/j.solener.2013.07.005

[18] Köntges M, Kurtz S, Packard CE, Jahn U, Berger K, Kato K, et al. Review of Failures of Photovoltaic Modules. IEA-Photovoltaic Power Systems Programme. 2014. 978-3-906042-16-9

[19] Singh J, Belmont J, Tamizhmani G. Degradation analysis of 1900 PV modules in a hot-dry climate: Results after 12 to 18 years of field exposure. Conf Rec IEEE Photovolt Spec Conf. 2013; 3270–3275.

[20] Mekhilef S, Saidur R, Kamalisarvestani M. Effect of dust, humidity and air velocity on efficiency of photovoltaic cells. Renew Sustain Energy Rev. 2012;16: 2920–2925. doi: 10.1016/j.rser.2012.02.012

[21] Gul RM, Ali M, Zafar FU, Noman M. The impact of static wind load on the mechanical integrity of different commercially available mono-crystalline photovoltaic modules. Eng Reports. 2020; 1–13. doi: 10.1002/eng2.12276

[22] Morlier A, Haase F, Kontges M. Impact of Cracks in Multicrystalline Silicon Solar Cells on PV Module Power -A Simulation Study Based on Field Data. IEEE J Photovoltaics. 2015;5: 1735–1741. doi: 10.1109/JPHOTOV.2015.2471076

[23] IEC. Terrestrial photovoltaic (PV) modules–Design qualification and type approval–Part 2: Test procedures. IEC 61215–2. 2016.

[24] Ali S, Ali A, Saher S, Agha HS, Bin Abdul Majeed H, Mahmood FI, et al. A comprehensive study of 18–19 years field aged modules for degradation rate determination along with defect detection and analysis using IR, EL, UV. Proceedings of 2018 15th International Bhurban Conference on Applied Sciences and Technology, IBCAST 2018. 2018.

[25] Ulfat I, Javed F, Abbasi FA, Kanwal F, Usman A, Jahangir M, et al. Estimation of solar energy potential for Islamabad, Pakistan. Energy Procedia. 2012;18: 1496–1500. doi: 10.1016/j.egypro.2012.05.166

[26] Chicca M, Tamizhmani G. Nondestructive Techniques to Determine Degradation Modes: Experimentation with 18 Years Old Photovoltaic Modules. 2015.

[27] Packard CE, Wohlgemuth JH, Kurtz SR. Development of a Visual Inspection Data Collection Tool for Evaluation of Fielded PV Module Condition. NREL Tech Rep. 2012.

[28] Fuyuki T, Kitiyanan A. Photographic diagnosis of crystalline silicon solar cells utilizing electroluminescence. Appl Phys A Mater Sci Process. 2009. doi: 10.1007/s00339-008-4986-0

[29] Kontges M, Morlier A, Eder G, Fleis E, Kubicek B, Lin J. Review: Ultraviolet Fluorescence as Assessment Tool for Photovoltaic Modules. IEEE J Photovoltaics. 2020;10: 616–633. doi: 10.1109/JPHOTOV.2019.2961781

[30] Pern FJ, Glick SH. Fluorescence analysis as a diagnostic tool for polymer encapsulation processing and degradation. 1994;573. doi: 10.1063/1.45730

[31] Morlier A, Siebert M, Kunze I, Blankemeyer S, Köntges M. Ultraviolet fluorescence of ethylene-vinyl acetate in photovoltaic modules as estimation tool for yellowing and power loss. 2018 IEEE 7th World Conf Photovolt Energy Conversion, WCPEC 2018—A Jt Conf 45th IEEE PVSC, 28th PVSEC 34th EU PVSEC. 2018; 1597–1602.

[32] Morlier A, Siebert M, Kunze I, Mathiak G, Kontges M. Detecting Photovoltaic Module Failures in the Field during Daytime with Ultraviolet Fluorescence Module Inspection. IEEE J Photovoltaics. 2017;7: 1710–1716. doi: 10.1109/JPHOTOV.2017.2756452

[33] Eder G, Knöbl K, Voronko Y, Grillberger P, Kubicek B. UV-Fluorescence measurements as tool for the detection of degradation effects in PV- Modules. 8th Eur Weather Symp. 2017; 1–7.

[34] Omazic A, Oreski G, Halwachs M, Eder GC, Hirschl C, Neumaier L, et al. Relation between degradation of polymeric components in crystalline silicon PV module and climatic conditions: A literature review. Sol Energy Mater Sol Cells. 2019;192: 123–133. doi: 10.1016/j.solmat.2018.12.027

[35] Chattopadhyay S, Dubey R, Kuthanazhi V, John JJ, Solanki CS, Kottantharayil A, et al. Visual degradation in field-aged crystalline silicon PV modules in India and correlation with electrical degradation. IEEE J Photovoltaics. 2014;4: 1470–1476. doi: 10.1109/JPHOTOV.2014.2356717

[36] Lin CC, Krommenhoek PJ, Watson SS, Gu X. Depth profiling of degradation of multilayer photovoltaic backsheets after accelerated laboratory weathering: Cross-sectional Raman imaging. Sol Energy Mater Sol Cells. 2016;144: 289–299. doi: 10.1016/j.solmat.2015.09.021 PMC13576181 42746450

[37] Czanderna AW, Emery KA, Dhere RG, Renewable N. Weathering degradation of eva encapsulant and the effect of its yellowing on solar cell efficiency. 1991; 557–561.

[38] Wohlgemuth JH, Kempe MD, Miller DC. Discoloration of PV Encapsulants. 2013; 3260–3265.

[39] Park NC, Jeong JS, Kang BJ, Kim DH. The effect of encapsulant discoloration and delamination on the electrical characteristics of photovoltaic module. Microelectronics Reliability. 2013. doi: 10.1016/j.microrel.2013.07.062

[40] Patel AP, Sinha A, Tamizhmani G. Field-Aged Glass/Backsheet and Glass/Glass PV Modules: Encapsulant Degradation Comparison. IEEE J Photovoltaics. 2020;10: 607–615. doi: 10.1109/JPHOTOV.2019.2958516

[41] Czanderna AW, Pern FJ. Encapsulation of PV modules using ethylene vinyl acetate copolymer as a pottant: A critical review. Sol Energy Mater Sol Cells. 1996. doi: 10.1016/0927-0248(95)00150-6

[42] Sinha A, Sastry OS, Gupta R. Nondestructive characterization of encapsulant discoloration effects in crystalline-silicon PV modules. Sol Energy Mater Sol Cells. 2016;155: 234–242. doi: 10.1016/j.solmat.2016.06.019

[43] Peike C, Philip H, Bl M, Schmid P, Weiß K, Michael K. Impact of Permeation Properties and Backsheet-Encapsulant Interactions on the Reliability of PV Modules. 2012;2012. doi: 10.5402/2012/459731

[44] Dechthummarong C, Wiengmoon B, Chenvidhya D, Jivacate C, Kirtikara K. Solar Energy Materials & Solar Cells Physical deterioration of encapsulation and electrical insulation properties of PV modules after long-term operation in Thailand. Sol Energy Mater Sol Cells. 2010;94: 1437–1440.

[45] Jordan DC, Wohlgemuth JH, Kurtz SR. Technology and Climate Trends in PV Module Degradation Preprint. 2012.

